# Gingival Zenith Correction by Laser Gingivectomy

**DOI:** 10.7759/cureus.51495

**Published:** 2024-01-01

**Authors:** Shylaja Mohan, Vamsi Lavu

**Affiliations:** 1 Periodontics and Implantology, Sri Ramachandra Institute of Higher Education and Research, Chennai, IND

**Keywords:** esthetics, gingivectomy, diode laser, interdisciplinary, gingival zenith

## Abstract

A healthy gingival structure showcases a knife-edged gingival margin, firmly adherent to the tooth surface, accompanied by a cone-shaped or pointed interdental papilla, mirroring the lowest point in the gingival margin, termed the gingival zenith. Tooth transposition denotes an anomaly in tooth positioning, commonly involving the canine and the first maxillary premolar. It represents a form of ectopic eruption, wherein two adjacent teeth interchange positions within the same quadrant of the dental arch. Laser wavelengths are utilized for precise incision of gingival tissues to address restorative, cosmetic, and periodontal needs. Post-operatively, rapid healing and diminished discomfort are frequently observed, often eliminating the necessity for periodontal packing or sutures. Gingivectomy is the accepted modality for the establishment of esthetics in situations with abnormal gingival contour. This study highlights the use of contemporary technology namely a 940 nm diode laser for correction of gingival zenith to achieve optimal esthetics post orthodontic treatment.

## Introduction

The anatomy of a smile is integral to dentistry, requiring meticulous examination of all oral elements. Crafting the ideal smile involves analyzing and evaluating the face, lips, gingival tissues, and teeth, appreciating how they collectively contribute to a harmonious appearance [1]. Symmetry and balance in facial and dental features are essential for achieving this ideal smile [2].

Recognizing that form follows function, special attention must be given to the anterior teeth, which play a vital role in oral health [3]. A comprehensive approach to diagnosing and treatment planning for esthetic cases is crucial for attaining a smile that not only enhances facial appearance but also promotes oral health. To predict the final esthetic outcome and ensure optimal results in gingival contour rehabilitation (including crown lengthening, implants, restorative procedures, and orthodontic therapy), it is imperative to consider gingival contours during treatment planning [1]. The gingival line, a significant aspect of gingival morphology, is defined as the line connecting the tangents of the gingival zeniths of the central incisor and canine, with the gingival zenith representing the most apical aspect of the free gingival margin [4]. The existing body of literature presents numerous methodologies employed to attain the goal of correcting altered gingival zenith following the transposition of the tooth as a part of orthodontic treatment. These encompass techniques such as soft tissue gingivectomy, crown exposure through a combination of soft and hard tissue resection, and orthodontic extrusion of the tooth [5]. A modernized approach to soft and hard tissue removal incorporates the use of lasers. The study discussed the use of a diode laser (940 nm wavelength, EZlase; Mumbai, India: Biolase) for correction of the gingival zenith of a transposed maxillary tooth by a gingivectomy procedure.

## Case presentation

**Figure 1:**
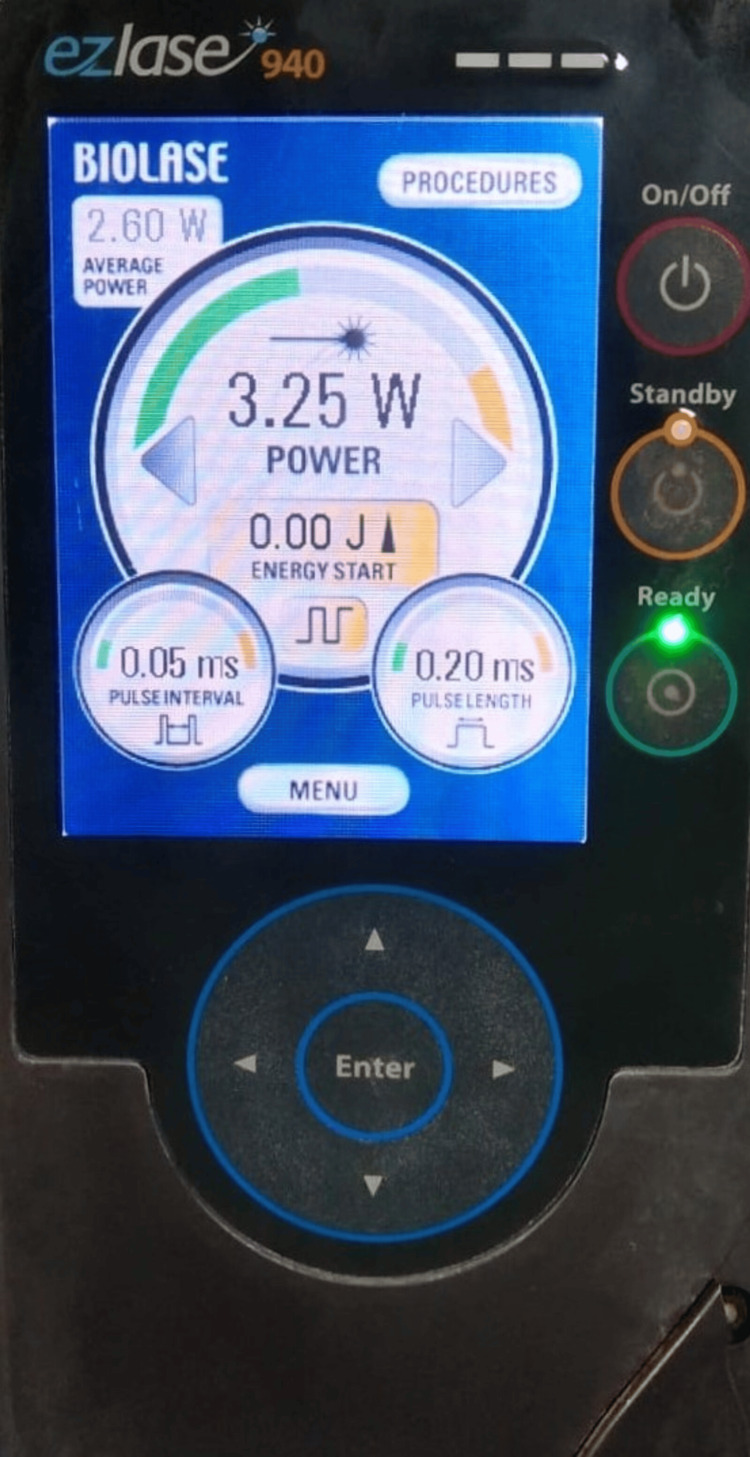
Biolase EZLase (Mumbai, India) 940 nm diode laser equipment.

A 19-year-old female patient undergoing fixed orthodontic therapy was referred from the department of orthodontics to the periodontics OPD for correction of gingival zenith following the transposition of the upper left canine and premolar. On examination, the gingival zenith in relation to the buccal aspect of transposed premolar in the 24 region was found to be more coronally located with a periodontal probing depth of more than 5 mm, width of keratinized gingiva of 7 mm on the mid-buccal aspect and a thick gingival biotype (Table 1). The location of the gingival zenith (the most apical point in the gingival marginal scallop) in the transposed premolar was lower than the adjacent lateral incisor. A repositioning of the gingival zenith by 3 mm was indicated to achieve a more optimal zenith position and better esthetics for the patient. Since there was an adequate width of keratinized gingiva and pocket depth of 5 mm gingivectomy up to 3 mm was planned with a 940-nm diode laser (operating parameters being 3.25 W, a pulse mode of 20 ms/20 ms-pulse duration/pulse interval 400 micro fiber-contact mode) (Figure 1).

**Table 1:** Mucogingival parameters for the tooth in the transposed site.

Tooth no.	Parameters	Mesio-buccal (mm)	Mid-buccal (mm)	Disto-buccal (mm)
24	Probing depth	5	5	4
	Width of keratinized gingiva	10	7	6
	Width of attached gingiva	5	2	2

**Figure 2:**
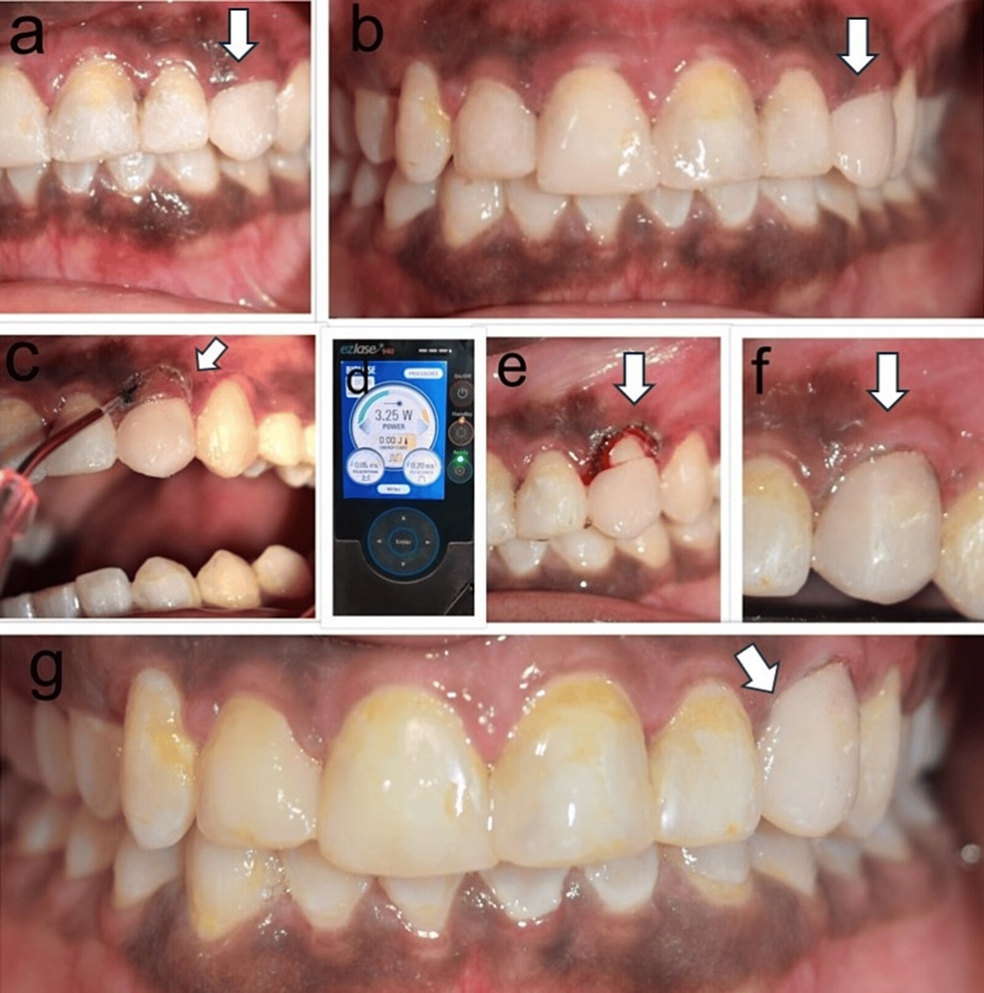
Pre-operative, intra-operative, and post-operative pictures of the gingivectomy procedure performed for correction of altered gingival zenith in the transposed tooth. The images (a and b) represent the pre-operative image of altered gingival zenith in the transposed 24 left upper first premolar, (c) intra-operative image of a gingivectomy performed with 940 nm diode laser in the transposed 24 left upper first premolar, (d) 940 nm diode EZLase (Mumbai, India: Biolase), 3.25 W, pulsed mode 20 ms pulse duration and 20 ms pulse interval with 400-micron fiber, (e) cervical wedge-shaped defect evident in relation to 24 left upper first premolar buccal aspects after gingivectomy, (f) cervical defect after restoration with light-cured composite material at two-week post-operative, and (g) post-operative image at three months follow-up with the new zenith being stable in relation to 24 left upper first premolar. Arrows indicate tooth 24, was subjected to gingivectomy by 940 nm diode laser (Biolase EZLase), and composite light cure restoration was performed.

A layer-by-layer cutting was performed in contact mode after initiating the tip. Cutting was initially performed with the tip at 90 degrees to the soft tissue. The beveled margin in the transposed premolar was created by changing the angulation to 30 degrees. Hemostasis was achieved as an incision was performed with the laser. The cuff of tissue was removed using a curette. A cervical wedge-shaped defect was observed in the transposed premolar and this was restored with a light cure composite (3M -Z350; St. Paul, MN: 3M). The wedge-shaped defect could have been the result of resorption during the tooth movement. The post-operative review was done at two weeks and three months (Figures 2a-2g).

## Discussion

The smile's esthetics are significantly influenced by the gingival zenith's positioning, identified as the most apical point in the gingival marginal scallop. Its spatial orientation in both the apico-coronal and mesiodistal dimensions holds significance. The ideal positioning entails the central incisor's zenith in the distal third, the lateral incisor centrally, and the canine ranging from the anterior to distal thirds [5]. The location of the gingival zenith is an important factor affecting the pink esthetics and often is used in restorative dentistry approaches, to establish esthetics in restorations especially when the individual has a high lip line. Two concepts, namely the super-normal and the natural, have been introduced. The super-normal concept aims to transcend natural norms based on patient preferences, while the natural concept seeks harmony with overall appearance by considering artistic determinants, personality, and anatomic norms [4].

The framing of teeth within this gingival framework significantly influences smile esthetics. Both excessive gum exposure (gummy smile) and significant recession can negatively impact the overall esthetics. The height of the gingival contour plays a crucial role in determining the beauty of a smile, affecting the axial inclination and emergence profile of the teeth. Even seemingly minor details, such as the position of the gingival zenith, can collectively influence the overall pleasing appearance of a smile [6].

Tooth transposition, an ectopic eruption condition, involves the unique interchange of positions between two permanent adjacent teeth within the same quadrant of the dental arch [7]. This condition can be categorized as complete, where the affected teeth entirely switch positions, or incomplete, with only the crowns being transposed while the roots maintain their normal position. In instances such as canine transposition with premolar or canine with lateral incisor, traditional treatment approaches have involved aligning the teeth in their transposed positions or extracting one or both transposed teeth [8]. Correcting tooth transposition poses challenges and requires extensive treatment duration. It necessitates considering not only root position and inclination but also the available bone volume at the site of tooth movement. It's crucial to manage mechanics carefully to avoid buccal bone plate damage, occlusal interferences, and root resorption [9].

Orthodontic treatment aims to restore proper occlusion, enhance facial aesthetics, and preserve periodontal, and tooth support structure health. However, in numerous cases, a multi-disciplinary clinical approach becomes essential to achieve all treatment objectives [10].

Gingivectomy in esthetic surgery is performed to reposition the gingival margin to match the zenith and increase crown height as preparation for restorative work like veneers, crowns, etc. [11]. While gingivectomy offers advantages in terms of simplicity, it is accompanied by drawbacks such as increased post-operative discomfort and risk of post-operative hemorrhage [12]. The diode lasers have demonstrated benefit in soft tissue surgery dental practice, and they have been integral in gingivectomy [13]. Laser provides potential advantages in terms of reduced pain compared to traditional methods, effective hemostasis, and minimized infection risks despite its disadvantage being expensive [10,14]. Additionally, they may facilitate soft tissue healing and enhance esthetic outcomes. This study explores the utilization of diode lasers for a gingivectomy in a novel situation involving the altered zenith of a transposed tooth.

In certain instances, a combination of therapies may be necessary, including orthognathic surgery [15], orthodontic treatment [16], lip repositioning [17], botulinum injection [18], and esthetic crown lengthening [6]. The selection and sequencing of treatments require careful consideration. Employing the proposed stepwise analysis, clinicians can visualize potential esthetic outcomes and help patients understand the anticipated benefits and side effects of each procedure beforehand [19]. Exploring alternative treatments may be advisable to optimize outcomes, enhance patient comfort, and ensure satisfaction.

This study focuses on the correction of altered gingival zenith due to transposition and adequate esthetics were restored with the use of a 940 nm diode laser with minimal risk of bleeding and pain.

## Conclusions

Assessment of gingival structure and esthetic criteria should be a standard component of orthodontic planning. Furthermore, fostering multidisciplinary collaboration, in the field of periodontics is crucial for enhancing treatment precision and addressing esthetic-related issues. Utilizing the diode lasers acts as an alternative approach in the precise management of correcting altered gingival zenith resulting from transposition as a course of orthodontic therapy.
